# Chewing and Cognitive Improvement: The Side Matters

**DOI:** 10.3389/fnsys.2021.749444

**Published:** 2021-12-23

**Authors:** Maria Paola Tramonti Fantozzi, Vincenzo De Cicco, Davide De Cicco, Paola d’Ascanio, Enrico Cataldo, Luca Bruschini, Ugo Faraguna, Diego Manzoni

**Affiliations:** ^1^Department of Translational Research and of New Surgical and Medical Technologies, University of Pisa, Pisa, Italy; ^2^Department of Neurosciences, Reproductive and Odontostomatological Sciences, University of Naples Federico II, Naples, Italy; ^3^Department of Physics, University of Pisa, Pisa, Italy; ^4^Department of Surgical, Medical and Molecular Pathology and Critical Care Medicine, University of Pisa, Pisa, Italy; ^5^Department of Developmental Neuroscience, IRCCS Fondazione Stella Maris, Pisa, Italy

**Keywords:** unilateral chewing, trigeminal asymmetry, pupil size, anisocoria, cognitive performance, locus coeruleus

## Abstract

Chewing improves cognitive performance, which is impaired in subjects showing an asymmetry in electromyographic (EMG) masseter activity during clenching. In these subjects, the simultaneous presence of an asymmetry in pupil size (anisocoria) at rest indicates an imbalance in Ascending Reticular Activating System (ARAS) influencing arousal and pupil size. The aim of the present study was to verify whether a trigeminal EMG asymmetry may bias the stimulating effect of chewing on cognition. Cognitive performance and pupil size at rest were recorded before and after 1 min of unilateral chewing in 20 subjects with anisocoria, showing an EMG asymmetry during clenching. Unilateral chewing stimulated performance mainly when it occurred on the side of lower EMG activity (and smaller pupil size). Following chewing on the hypotonic side, changes in cognitive performance were negatively and positively correlated with those in anisocoria and pupil size, respectively. We propose that, following chewing on the hypotonic side, the arousing effects of trigeminal stimulation on performance are enhanced by a rebalancing of ARAS structures. At variance, following chewing on the hypertonic side, the arousing effect of trigeminal stimulation could be partially or completely prevented by the simultaneous increase in ARAS imbalance.

## Introduction

Recent investigations have shown how chewing can exert a stimulating effect on cognitive performance. Gum chewing enhances alertness and attention (Tucha et al., 2004; Allen and Smith, 2012; Johnson et al., 2012), speed of neural processing (Hirano et al., 2013), learning and memory (Allen et al., 2008; Smith, 2009). Shortening of reaction times and latencies of stimulus-triggered evoked potentials are also observed (Sakamoto et al., 2009; Hirano and Onozuka, 2014). The decrease in reaction time (Hirano and Onozuka, 2014) and the enhancement of short-term memory processing (Hirano et al., 2008) induced by chewing are coupled to an increase in the blood-oxygen-level dependent (BOLD) signal in those brain regions activated by a cognitive task. Stimulation of cognitive performance elicited by chewing can last for a period of 15–20 min (Onyper et al., 2011) and even more, when hard pellet is chewed (Tramonti Fantozzi et al., 2017). A reduced pellet consistency elicited weaker (Tramonti Fantozzi et al., 2017) or no effects on cognitive performance (Davidson, 2011).

These stimulating effects of chewing could be attributed to the trigeminal influences on the Ascending Reticular Activating System (ARAS), described by Moruzzi and Magoun (1949), which controls arousal and attention. Within this complex and multifaceted structure, the noradrenergic Locus Coeruleus (LC) neurons (De Cicco et al., 2018) have been particularly highlighted for their major contribution to the control of cognitive performance (Aston-Jones and Cohen, 2005). It is known that the trigeminal afferents impinge upon several ARAS structures, including the reticular formation, the pedunculopontine and laterodorsal tegmental nuclei, the histaminergic neurons in the tuberomammillary nucleus and the LC (De Cicco et al., 2018). Recent investigations have shown that chewing-induced changes in cognitive performance are positively correlated with the corresponding changes observed in task-related mydriasis (Tramonti Fantozzi et al., 2017), which is one important indicator of arousal and autonomic activation (Bradshaw, 1967; Bradley et al., 2008). This finding indicates that trigeminal pathways involved in cognitive improvement have also access to the neuronal networks controlling pupil size during the task. There are several cortical and subcortical regions whose activity is somehow linked to or whose stimulation elicits behavioral changes in pupil size (Reimer et al., 2016; Peinkhofer et al., 2019; Cazettes et al., 2021). Three of these structures belong to ARAS (the raphe nuclei, the cholinergic system, and the LC) and two of them receive also trigeminal afferents (the cholinergic system and the LC) (De Cicco et al., 2018). It is of interest that the rapid dilatations of pupil size during rest and locomotion are more strongly coupled to the norepinephrine than the acetylcholine release at cortical level (Reimer et al., 2016). In both monkey (Joshi et al., 2016) and rat (Liu et al., 2017) micro stimulation of LC elicits pupil dilatation; moreover, an impressive covariation of LC activity and pupil size (Rajkowski et al., 1994; Murphy et al., 2014; Joshi et al., 2016) was found in both human and animals (Joshi and Gold, 2020), suggesting the latter as a proxy of the central noradrenergic system activity (Alnæs et al., 2014; Mathôt, 2018). Finally, the relation between LC activity and cognitive control/performance has been well documented (Aston-Jones and Cohen, 2005; Grueschow et al., 2020). Although the topic is far from being definitively assessed, the LC seems to be the best candidate for mediating the trigeminal effects on cognitive performance.

Moreover, recent investigations have shown that an asymmetry in electromyographic (EMG) masseter activity during clenching is detrimental for cognitive performance (Tramonti Fantozzi et al., 2017) and is associated to a pupil asymmetry (anisocoria). A very significant correlation was observed between anisocoria and EMG asymmetry, pupil size being larger on the side of higher EMG activity (hypertonic side). Correction of the trigeminal imbalance by an orthotic splint leads to a great reduction of the anisocoria, with an associated improvement in performance. These results could be explained by a trigeminal-induced imbalance at the ARAS level, leading to an asymmetric cortical excitability, which disrupts cognitive performance. It has been shown, in fact, that the cortical imbalance elicited by a unilateral lesion can be more detrimental for cognitive performance with respect to a double symmetric lesion (Lomber and Payne, 1996).

Considering these findings, one may wonder whether and how the stimulating effects of chewing may interact with a basal asymmetry in sensorimotor trigeminal activity coupled to anisocoria.

In order to address this issue, we studied subjects showing a right or left predominance of masseter EMG activity during clenching. In this population we tested whether the stimulating effect of unilateral chewing on cognitive performance was affected by the side (hypertonic or hypotonic) where this activity was carried out.

## Materials and Methods

### Subjects

A randomly recruited population of 30 individuals (15 females), free from metabolic, endocrine, neurological and psychiatric diseases, were considered for this study, which was approved by the Ethical Committee of the Pisa University (Unipi Bioethical Committee No: 12-2019). Twenty-two of them (15 females) showed a masseter EMG asymmetry during clenching greater than 20% of the corresponding mean [(EMG hypertonic-EMG hypotonic side)/(mean of the two sides)]. All of them were natural right-handers. Twenty subjects (age 35.2 ± 12.6, 20–54 years, 13 females) showing EMG asymmetry could be enrolled in the study. When the muscles were relaxed and the arches slightly apart, all the 20 subjects showed an anisocoria at rest higher than 0.1 mm. They were divided, according to the side of greater EMG activity during clenching in right (*n* = 10, 8 females) and left (*n* = 10, 5 females) dominant subjects (Clenching Dominance). Among them, 13 had full dentition without any masticatory dysfunctions and 5 showed loss of 1–4 molar teeth. Signs of temporo-mandibular joint (TMJ) dysfunction were observed in 3 subjects (one of them showing also a molar loss). In all these subjects, the sides of higher and lower EMG activity will be referred to as the hypertonic and the hypotonic side, respectively. In all instances, pupil size at rest was larger on the side of higher EMG activity during clenching.

### Workflow of the Experiments

**FIGURE 1:**
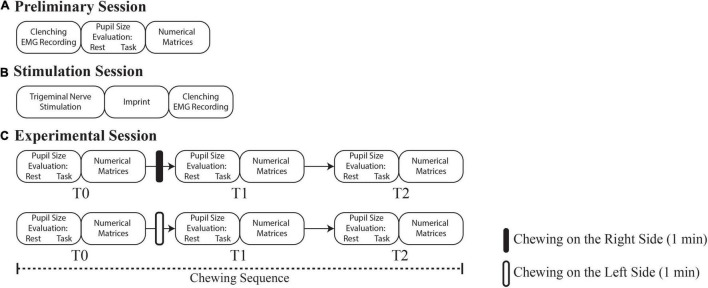
Flowchart of the experiments. **(A)** Preliminary session, time 0 days. **(B)** Stimulation session, time 18–24 days. **(C)** Experimental session, time 80–86 days. Rounded corners rectangles indicate the pupil size/performance measurements and the experimental procedures operated in each session. T1 and T2 correspond to 0 and 30 min soon after the period of unilateral chewing, respectively.

In a preliminary session (Figure 1A) the following evaluations were performed:

a) Left and right masseter EMG activity during clenching,
b) Bilateral measurement of pupil size at rest and
c) During the execution of a haptic, Tangram-based task,
d) Performance assessment in a cognitive task, based on the Spinnler-Tognoni numeric matrices test (Tramonti Fantozzi et al., 2017).

About 18 days after the preliminary session, an electrical trigeminal nerve stimulation was performed to induce masticatory muscles relaxation (stimulation session, see Figure 1B). Soon after stimulation, the occlusal condition obtained by bringing the arches into contact was captured by an imprint. The latter normalized the abnormal teeth contacts and, when subjects were clenching with the imprint in their mouth, the EMG asymmetry observed in the initial session was abolished or greatly reduced. This imprint was utilized for manufacturing a bite splint (2 weeks required) that the subject wore for 2 weeks more before performing a new EMG evaluation during clenching with the bite interposed between the dental arches (not shown in Figure 1). This procedure documented the bite ability to reduce the occlusal imbalance.

Following an overall period of 48–54 days of bite wearing, the experimental session began with a baseline evaluation (T0) of pupil size (b, c) and performance parameters (d). Pupil size measurements were performed while subjects kept the arches slightly apart, in the normal resting position. These evaluations took about 5 min to be completed (Figure 1C, upper row).

Soon after, subjects were invited to unilaterally chew a custom-made hard pellet (see the section “Chewed Pellet”) for 1 min on the right side. The pellet, left on the table in front of the subject was self-administered. The initial pupil size and performance measurements were repeated soon after 1 min period of unilateral chewing, i.e., approximatively 6 min (T1) following T0, and 30 min later (T2). After 1 h the entire T0-T1-T2 sequence (hereby referred to as “chewing sequence”) was repeated (Figure 1C, lower row), inviting the subjects to chew another pellet (identical to the first one) on the left side. The subjects were invited to rest between the T1 and T2, as well as between chewing sequences.

### Evaluated Parameters

The analysis was based on the following parameters.

1) Pupil size-related parameters:
a) Left and right pupil size at rest;
b) Changes in left and in right pupil size during the haptic task (task-related mydriasis);
c) Difference in pupil size between the two sides (anisocoria).
2) Performance parameters (from the numerical matrices test):
d) Performance Index (PI: target numbers retrieved in 15 s/15);
e) Scanning Rate (SR: target + non-target numbers scanned in 15 s/15);
f) Error Rate (ER: missed target numbers + non-target numbers wrongly underlined/15).

All metrics are described in detail elsewhere (Tramonti Fantozzi et al., 2017).

### Numerical Matrices Test

Subjects had to sequentially scan three numerical matrices made of ten lines and ten columns, with the goal of retrieving and tick with a pencil as many of the target numbers indicated above each matrix, as described elsewhere (Tramonti Fantozzi et al., 2017). The position of target numbers in the matrices presented at T0, T1, and T2 changed in order to prevent learning processes.

### Electrical Trigeminal Nerve Stimulation for Bite Splint Manufacturing

Both trigeminal mandibular branches were stimulated by two couples of electrodes (IACER, I-Tech Medical Division, stimulating surface of each electrode: 164 mm^2^) positioned, respectively on the incisura sigmoidea and on the submental triangle and connected with two independent I.A.C.E.R stimulators (Martellago, Venice, Italy). Biphasic (cathodal/anodal) current pulses (0.54 msec, 21–25 mA) were delivered at the frequencies of 0.618 Hz (incisura sigmoidea) and of 40 Hz (submental triangle) leading to contraction/relaxation of masseters and to sustained activation of lowering muscles, triggering small amplitude (1 mm) mandibular movements in the sagittal plane. The current intensity on each side was adjusted so to obtain symmetric EMG responses.

### Haptic Task

During the haptic task, subject’s head was restrained within the view-impeding pupillometric device. The experimenter extracted from the Tangram puzzle the parallelogram-shaped piece and put it in the subject’s right (dominant) hand. The task consisted in positioning the parallelogram back into the puzzle frame: pupil size recording was performed at the beginning of puzzle exploration. Further experimental details are described elsewhere (Tramonti Fantozzi et al., 2017).

### Chewed Pellet

The hard pellet (OCM Projects, Italy), cylindrical in shape (1.0 cm × 1.0 cm × 1.5 cm), gray in color, sugar free, odor, and tasteless, was made of a silicon rubber (gls50, Prochima, Italy) with a reticular structure and a constant (not modified by chewing) hardness of 60 Shore OO. The spring constant of this material, that quickly recovered its original shape following a deformation, was 15.7 N/m.

### Data Acquisition

A corneal topographer-pupillographer (MOD i02, with chin support, CSO, Florence, Italy) was utilize in order to measure pupil size, in constant artificial lighting of 40 lux (photopic condition), during fixation of a light spot displayed by the instrument. Pupil size was acquired by a CCD camera sensor (working distance: 56 mm, acquisition time: 33 msec). Measurements were separately taken for both eyes.

Duo-trode surface Ag/AgCl electrodes (interelectrode distance 19,5 mm, MyoTronics, Seattle, WA, United States) recorded EMG activity during clenching for 2–3 s (De Cicco et al., 2014). EMG was sampled at 720 Hz by a K6-I MyoTronics system, high-pass (cutoff frequency: 15 Hz) and notch (50 Hz) filtered, full-wave rectified and displayed on a monitor, together with the mean value obtained during the EMG burst.

### Statistical Analysis

The comparison of baseline (T0) performance parameters (PI, SR, ER), left and right pupil size, task-related mydriasis and anisocoria (taken as absolute value) between left and right dominant subjects were performed by *t*-test for independent data.

In order to simplify data description, the effects of chewing on performance and pupil size parameters were assessed with respect to the sides of higher and lower EMG activity, that will be indicated as the hypertonic and the hypotonic side, respectively. For this purpose, a 2 Chewing Side (chewing on hypertonic side, chewing on hypotonic side) x 3 Time (T0, T1, T2) repeated measures ANOVA, with Clenching Dominance (right, left) as a between-subjects factor was applied. Pupil size at rest and task-related mydriasis were evaluated separately for both the hypertonic and the hypotonic side, while anisocoria was taken with its absolute value. Gender-related differences were addressed by a 2 Chewing Side (chewing on hypertonic side, chewing on hypotonic side) x 3 Time (T0, T1, T2) repeated measures ANOVA, with Gender (males, females) as a between-subjects factor. Moreover, differences obtained at T1 with respect to T0 were compared by *t*-test across Chewing Side and between Clenching Dominance. When data distribution was significantly deviating from sphericity, *p*-values were corrected as appropriate.

Possible correlations between changes in pupil size (on the hypertonic and hypotonic side), anisocoria and performance parameters for both chewing conditions, were evaluated by linear regression analysis.

For each parameter the differences between T1-T0, T2-T1, and T2-T0 were evaluated for the chewing sequences performed on both the hypertonic and the hypotonic side. In this way, each subject contributed to the regression analysis with a total of six points.

Statistical Package for Social Sciences (IBM Corp. Released 2011. IBM SPSS Statistics for Windows, Version 20.0. Armonk, NY: IBM Corp^1^) was used for the analysis. Significance was set at *p* < 0.05.

## Results

### Outline of the Results

In the description of the results, we will first address the differences in baseline values of right and left pupil size/task-related mydriasis, anisocoria and performance parameters between the two groups with different clenching dominance (right, left). Then we will describe the effects of unilateral chewing (at the hypertonic and hypotonic side) on performance, pupil size, anisocoria and task-related mydriasis. Finally, correlations between changes in pupil size, anisocoria and performance parameters will be considered.

### Differences Between Left and Right Dominant Subjects in Baseline (T0) Parameters

**FIGURE 2:**
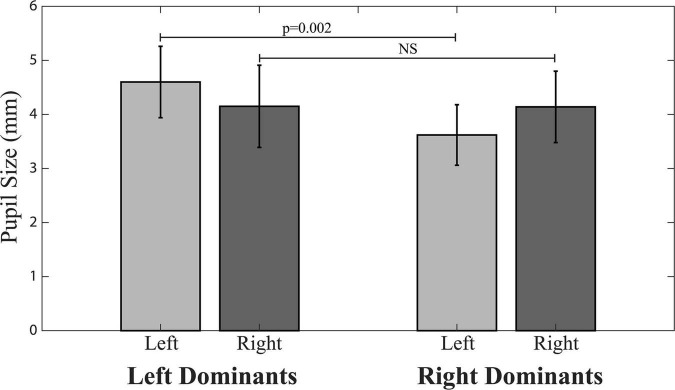
Pupil size in right and left dominant subjects.

The only differences found between left and right dominant subjects in baseline (average T0 values) parameters were those of ER and left pupil size. ER was lower in left (0.064 ± 0.041 Nos./s) than right dominant subjects (0.204 ± 0.15 Nos./s, *p* = 0.010). Left pupil size values were significantly larger in left (4.60 ± 0.66 mm) than right dominant subjects (3.62 ± 0.56 mm, *p* = 0.002), while right side pupil size did not differ between right (4.14 ± 0.66 mm) and left (4.15 ± 0.76 mm) dominant subjects (Figure 2).

### Effects of Unilateral Chewing on Performance-Related Parameters

**FIGURE 3:**
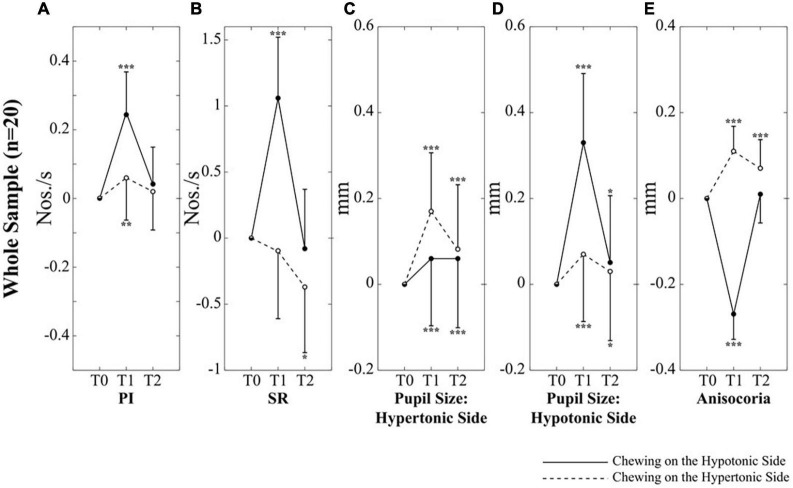
Changes in performance, pupil size and anisocoria elicited by unilateral chewing within the whole population. Changes in PI **(A)**, SR **(B)**, pupil size on the hypertonic **(C)** and hypotonic **(D)** side, as well as in anisocoria **(E)** are expressed as differences between T1/T2 and T0 values. Continuous and dotted lines refer to changes elicited by chewing on the hypotonic and hypertonic side, respectively. Asterisks refer to significant differences with respect to T0. *p < 0.05, **p < 0.01, and ***p < 0.005. Error bars represent SE of the mean.

Investigating the effects of chewing on the different sides in the two populations of subjects with left and right clenching dominance, a 2 Chewing Side (Hypertonic, Hypotonic) x 3 Time (T0, T1, T2) repeated measures ANOVA, with Clenching Dominance (right, left) as between-subjects factor was run. This analysis revealed significant Time, Chewing Side and Chewing Side x Time effects, for both PI and SR. All these effects arose from the significant Chewing Side x Time interactions (PI: *F*_(2,36)_ = 26.913, *p* < 0.0005; SR: *F*_(2,36)_ = 17.828, *p* < 0.0005) decomposed in Table 1 for both PI and SR. These results are also summarized in Figures 3A,B, where data have been represented as differences with respect to T0 values.

**TABLE 1:** Average ± SD of Performance Index (PI) and Scanning Rate (SR) values obtained before (T0) and after (T1, T2) unilateral chewing on both sides for the whole population as well as for the two populations of left and right dominants subjects.

Group	Chewing side	Performance index (PI)						Scanning rate (SR)					
		T0	T0 vs. T1	T1	T1 vs. T2	T2	T2 vs. T0	T0	T0 vs. T1	T1	T1 vs. T2	T2	T2 vs. T0
All subjects	Hypertonic	1.77 ± 0.53	*p* = 0.006	1.83 ± 0.55	NS	1.79 ± 0.52	NS	12.76 ± 2.09	NS	12.66 ± 2.28	*p* = 0.029	12.39 ± 2.22	*p* = 0.012
*N* = 20	Hypotonic	1.78 ± 0.53	*p* < 0.0005	2.03 ± 0.53	*p* < 0.0005	1.82 ± 0.49	NS	12.59 ± 2.02	*p* < 0.0005	13.65 ± 2.06	*p* < 0.0005	12.51 ± 2.01	NS
Right dominants	Hypertonic (right)	1.74 ± 0.48	NS	1.75 ± 0.49	NS	1.75 ± 0.50	NS	12.83 ± 1.91	NS	12.54 ± 2.23	NS	12.46 ± 2.16	NS
*N* = 10													
	Hypotonic (left)	1.73 ± 0.48	*p* < 0.0005	2.02 ± 0.52	*p* = 0.008	1.83 ± 0.44	*p* = 0.016	12.69 ± 1.76	*p* = 0.003	13.86 ± 1.94	*p* = 0.005	12.80 ± 1.76	NS
Left dominants	Hypertonic (left)	1.81 ± 0.59	*p* = 0.001	1.90 ± 0.62	NS	1.84 ± 0.56	NS	12.68 ± 2.36	NS	12.78 ± 2.44	*p* = 0.002	12.33 ± 2.4	*p* = 0.009
*N* = 10	Hypotonic (right)	1.82 ± 0.60	*p* < 0.0005	2.05 ± 0.57	*p* < 0.0005	1.81 ± 0.55	NS	12.50 ± 2.34	*p* < 0.0005	13.44 ± 2.26	*p* < 0.0005	12.21 ± 2.30	*p* = 0.032

**FIGURE 4:**
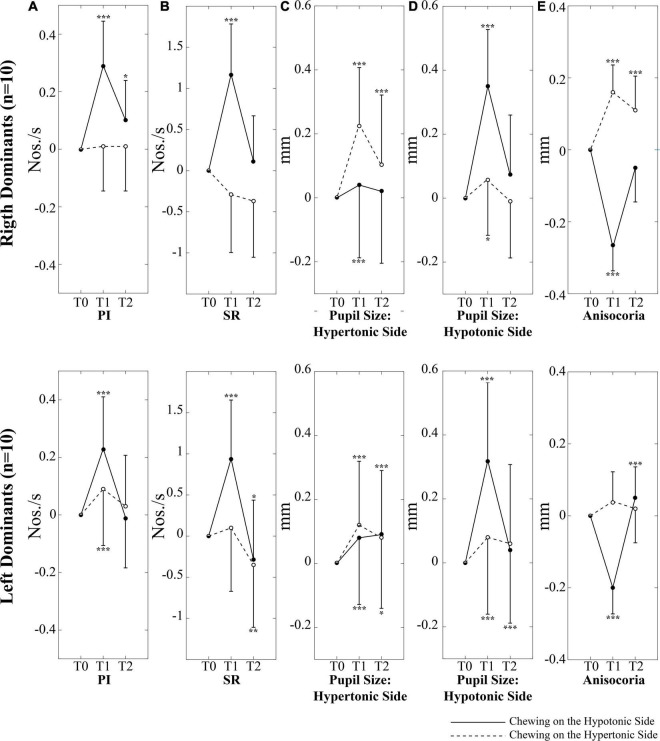
Changes in performance, pupil size, and anisocoria elicited by unilateral chewing among right and left dominant subjects. Changes in PI **(A)**, SR **(B)**, pupil size on the hypertonic **(C)** and hypotonic **(D)** side, as well as in anisocoria **(E)** are expressed as differences between T1/T2 and T0 values. Upper and lower rows represent right and left dominant subjects, respectively. Continuous and dotted lines refer to changes elicited by chewing on the hypotonic and hypertonic side, respectively. Asterisks refer to significant differences with respect to T0. *p < 0.05, **p < 0.01, and ***p < 0.005. Error bars represent SE of the mean.

When these interactions were analyzed, it appeared that chewing on both sides led to a significant increase in PI at T1; this effect, however, was stronger when chewing occurred on the hypotonic side (Figure 3A). Consistently, the change in PI at T1 with respect to T0 corresponded to 0.26 ± 0.12 Nos./s on the hypotonic side and to only 0.05 ± 0.08 Nos./s on the hypertonic side (*p* < 0.0005). All the effects of unilateral chewing on PI were lost at T2 (Figure 3A and Table 1). As shown in Figure 4A and Table 1, only minor differences in PI could be observed between left and right dominant subjects. A significant Chewing Side x Time x Clenching Dominance interaction could be found (*F*_(2,36)_ = 3.341, *p* = 0.047) since chewing on the hypertonic side increased this parameter exclusively among the left dominant subjects.

As to the SR, it was increased only when chewing occurred on the hypotonic side (*p* < 0.0005), while chewing on the hypertonic side led to a slight decrease in this parameter (Figure 3B). When data obtained at T2 were considered, it appeared that the SR increment observed following chewing on the hypotonic side was lost at T2, while the decrease elicited by chewing on the hypertonic side became more prominent (Figure 3B and Table 1). No significant differences could be observed between right and left dominant subjects.

Error rate values were independent upon Chewing Side, but showed significant Clenching Dominance (*F*_(1,18)_ = 8.346, *p* = 0.010) and Time (*F*_(2,36)_ = 6.699, *p* = 0.003) effects, since ER was larger in right than left dominant subjects (see the section “Differences Between Left and Right Dominant Subjects in Baseline (T0) Parameters”) and in the whole sample it decreased progressively from T0 (0.17 ± 0.16 Nos./s) to T1 (0.13 ± 0.12 Nos./s, *p* = 0.039) and to T2 (0.10 ± 0.13 Nos./s, *p* = 0.006).

No Gender-related effects and interactions could be found, with the exception of a Gender effect for SR (*F*_(1,18)_ = 5.404, *p* = 0.032), whose average value was larger in females (13.47 ± 2.12 Nos./s) than in males (11.44 ± 1.21 Nos./s).

### Effects of Unilateral Chewing on Pupil Size, Anisocoria, and Task-Related Mydriasis

A 2 Chewing Side (Hypertonic, Hypotonic) x 3 Time (T0, T1, T2) repeated measures ANOVA, with Clenching Dominance (right, left) as between-subjects factor was run for pupil size at rest and task-related mydriasis. The test was separately applied to data relative to the hypertonic and the hypotonic side. Significant Chewing Side, Time and Chewing Side x Time effects were found for the resting size of both pupils: similar to what observed for performance parameters, the two former effects arose from the significant Chewing Side x Time interaction (hypertonic side pupil: *F*_(2,36)_ = 16.633, *p* < 0.0005; hypotonic side pupil: *F*_(2,36)_ = 59.717, *p* < 0.0005) decomposed in Table 2 for both pupils. At variance no significant effects and interactions were observed for task-related mydriasis.

**TABLE 2:** Average ± SD of left and right pupil size values obtained before (T0) and after (T1, T2) unilateral chewing on both sides for the whole sample and for left and right dominant subjects.

Group	Chewing side	Pupil side	Pupil size					
			T0	T0 vs. T1	T1	T1 vs. T2	T2	T2 vs. T0
All subjects	Hypertonic	Hypertonic	4.32 ± 0.69	*p* < 0.0005	4.49 ± 0.61	*p* = 0.006	4.40 ± 0.68	*p* < 0.0005
*N* = 20		Hypotonic	3.79 ± 0.70	*p* < 0.0005	3.86 ± 0.70	*p* = 0.008	3.82 ± 0.72	*p* = 0.033
	Hypotonic	Hypertonic	4.30 ± 0.72	*p* < 0.0005	4.36 ± 0.72	NS	4.36 ± 0.72	*p* < 0.0005
		Hypotonic	3.83 ± 0.70	*p* < 0.0005	4.16 ± 0.71	*p* < 0.0005	3.88 ± 0.70	*p* = 0.028
Right dominants	Hypertonic (right)	Hypertonic	4.08 ± 0.64	*p* < 0.0005	4.31 ± 0.56	*p* = 0.002	4.18 ± 0.62	*p* = 0.001
*N* = 10		Hypotonic	3.54 ± 0.56	*p* = 0.036	3.60 ± 0.55	*p* = 0.028	3.53 ± 0.56	NS
	Hypotonic (left)	Hypertonic	4.07 ± 0.73	*p* = 0.003	4.11 ± 0.72	*p* = 0.030	4.09 ± 0.71	NS
		Hypotonic	3.55 ± 0.52	*p* < 0.0005	3.90 ± 0.56	*p* < 0.0005	3.62 ± 0.60	NS
Left dominants	Hypertonic (left)	Hypertonic	4.55 ± 0.68	*p* = 0.001	4.67 ± 0.63	NS	4.63 ± 0.70	*p* = 0.012
*N* = 10		Hypotonic	4.04 ± 0.76	*p* < 0.0005	4.12 ± 0.76	NS	4.10 ± 0.78	*p* < 0.0005
	Hypotonic (right)	Hypertonic	4.53 ± 0.67	*p* < 0.0005	4.61 ± 0.66	NS	4.62 ± 0.65	*p* < 0.0005
		Hypotonic	4.10 ± 0.77	*p* < 0.0005	4.42 ± 0.77	*p* < 0.0005	4.14 ± 0.72	NS

It appeared that chewing always increased the size of both pupils at T1 and this effect persisted to some extent at T2 (Figures 3C,D), whatever the chewing side (hypertonic, hypotonic) could be. However, the effect was always stronger on the ipsilateral pupil (Figures 3C,D). Consistently, when the differences between T1 and T0 were evaluated, chewing on the hypertonic side increased the size of the ipsilateral pupil by 0.17 ± 0.09 mm and that of the contralateral one by 0.07 ± 0.06 mm (*p* = 0.001), while chewing on the hypotonic side increased the ipsilateral pupil by 0.33 ± 0.08 mm and the contralateral one by 0.06 ± 0.03 mm (*p* = 0.001). Comparison of Figures 3C,D allows to appreciate the differences in the effects of hypotonic and hypertonic side chewing. The effects on the ipsilateral pupil were significantly larger when chewing occurred on the hypotonic side (*p* < 0.0005, compare Figure 3C, dotted line, with Figure 3D, continuous line). At variance, the effects of chewing on the contralateral pupil were similar for both chewing sides (compare Figure 3C, continuous line with Figure 3D, dotted line). Differences between right and left dominant subjects can be appreciated in Figure 4 and in Table 2. It has to be reported that a significant Chewing Side x Time x Clenching Dominance interaction (*F*_(2,36)_ = 7.237, *p* = 0.002) was found for the hypertonic side pupil. For this pupil, the larger increase in size elicited by ipsilateral chewing was found only among right dominant subjects (Figure 4C, upper row; chewing on the hypertonic, right side: 0.23 ± 0.08 mm; chewing on the hypotonic side: 0.04 ± 0.03 mm, *p* = 0.001), while values observed among left dominant subjects were comparable (Figure 4C, lower row; chewing on the hypertonic, left side: 0.12 ± 0.08 mm; chewing on the hypotonic side: 0.08 ± 0.02 mm, NS).

The pattern of changes observed for pupil size at the hypertonic and hypotonic side elicited modification in anisocoria. Also for anisocoria significant Chewing Side, Time and Chewing Side x Time effects were found, all of them arising from a significant Chewing Side x Time interaction (*F*_(2,36)_ = 79.982, *p* < 0.0005), decomposed in Table 3. This result arose since anisocoria increased at T1 after chewing on the hypertonic side and decreased following chewing on the hypotonic side (Figure 3E). At T2, the former effect tended to persist, while the latter was lost (Figure 3E and Table 2).

**TABLE 3:** Average ± SD of anisocoria values obtained before (T0) and after (T1, T2) unilateral chewing on both sides for all the subjects as well as for left and right dominants subjects.

Group	Chewing side	Anisocoria					
		T0	T0 vs. T1	T1	T1 vs. T2	T2	T2 vs. T0
All subjects	Hypertonic	0.52 ± 0.27	*p* = 0.001	0.63 ± 0.26	NS	0.59 ± 0.30	*p* = 0.005
*N* = 20	Hypotonic	0.47 ± 0.30	*p* < 0.0005	0.20 ± 0.26	*p* < 0.0005	0.48 ± 0.30	NS
Right dominants	Hypertonic (right)	0.54 ± 0.28	*p* = 0.001	0.70 ± 0.24	NS	0.65 ± 0.30	*p* = 0.001
*N* = 10	Hypotonic (left)	0.54 ± 0.28	*p* < 0.0005	0.27 ± 0.21	*p* = 0.008	0.49 ± 0.30	NS
Left dominants	Hypertonic (left)	0.51 ± 0.28	NS	0.55 ± 0.26	NS	0.53 ± 0.30	NS
*N* = 10	Hypotonic (right)	0.43 ± 0.28	*p* < 0.0005	0.23 ± 0.23	*p* < 0.0005	0.48 ± 0.28	*p* = 0.003

A significant Chewing Side x Time x Clenching Dominance interaction (*F*_(2,36)_ = 6.460, *p* = 0.009) was also found, since the increase in anisocoria at T1 was significant only among right dominants subjects (Figure 4E, upper row).

Finally, no significant Gender-related effects or interactions could be found for pupil size and task-related mydriasis on both sides, as well as for anisocoria.

### Correlations Between Changes in Performance and Changes in Pupil Size and Anisocoria

**FIGURE 5:**
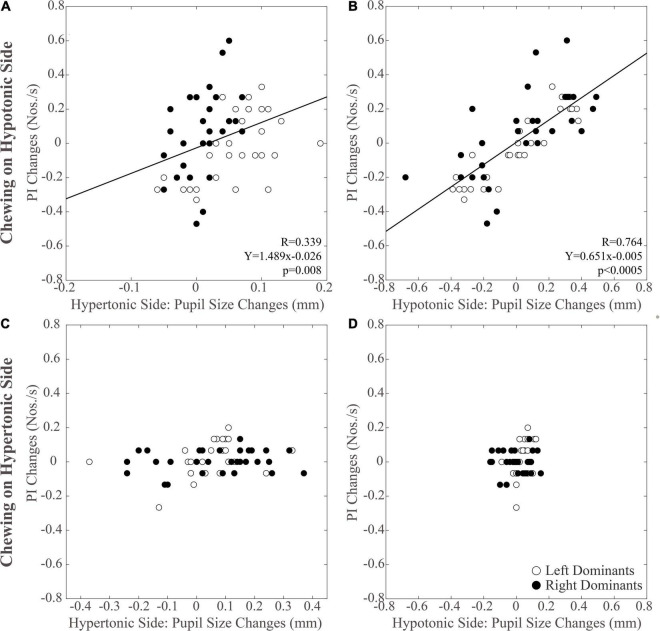
Relation between changes in PI and pupil size. Scatterplots of the changes in PI and in pupil size values observed on the hypertonic **(A,C)** and the hypotonic **(B,D)** sides, following unilateral chewing on the hypotonic **(A,B)** and the hypertonic **(C,D)** side. The regression lines refer to all the plotted points. In each graph, the black dots and the open dots represent the right and the left dominant subjects, respectively.

**FIGURE 6:**
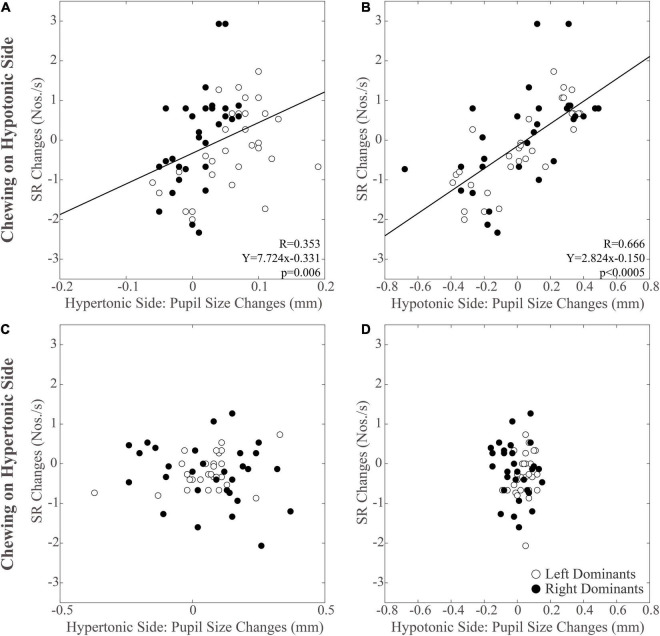
Relation between changes in SR and pupil size. Scatterplots of the changes in SR and in pupil size values observed on the hypertonic **(A,C)** and the hypotonic **(B,D)** sides, following unilateral chewing on the hypotonic **(A,B)** and the hypertonic **(C,D)** side. The regression lines refer to all the plotted points. In each graph, the black dots and the open dots represent the right and the left dominant subjects, respectively.

**FIGURE 7:**
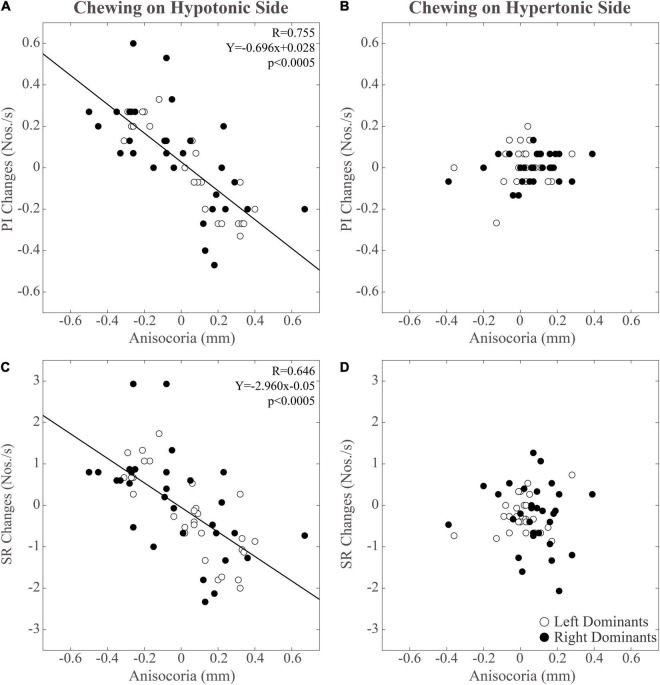
Relation between changes in performance-related parameters (PI, SR) and anisocoria. Changes in PI have been displayed in panels **(A,B)**. Changes in SR can be found in panels **(C,D)**. Data relative to unilateral chewing on the hypotonic and hypertonic side are shown in panels **(A,C)** and **(B,D)**, respectively. The regression lines refer to all the plotted points. In each graph, the black dots and the open dots represent the right and the left dominant subjects, respectively.

As shown in Figure 5, modifications in PI observed between the different time points were positively correlated with those in pupil size during chewing on the hypotonic (Figures 5A,B), but not on the hypertonic side (Figures 5C,D). This correlation was found for both the hypertonic (Figure 5A) and the hypotonic side (Figure 5B) pupils. Like PI changes, also SR changes were correlated to those in both hypertonic and hypotonic side pupils when chewing occurred on the hypotonic (Figures 6A,B), but not on the hypertonic side (Figures 6C,D). The performance changes elicited during chewing sequences on the hypotonic side were also negatively correlated with anisocoria changes (Figures 7A,C), while this was not the case for chewing sequences on the hypertonic side (Figures 7B,D). For performing this analysis, anisocoria was evaluated as the difference in pupil size between the hypertonic and the hypotonic side.

It must be pointed out that no significant correlations could be found between the chewing-induced modifications in pupil size, anisocoria and performance observed at T1 and the EMG asymmetry observed during clenching.

### Summary of the Main Observations

In conclusion, unilateral chewing could improve cognitive performance only when occurring on the hypotonic side, simultaneously reducing pupil asymmetry at rest. The reduction in anisocoria were joined to a bilateral increase in pupil size which was larger on the ipsilateral (hypotonic) side. The correlative analysis showed that changes in performance could be well predicted by changes in pupil size and anisocoria, but only when the masticatory activity occurred on the hypotonic side.

## Discussion

### Chewing on the Hypertonic and Hypotonic Side: Effects on Performance, Pupil Size, and Anisocoria

The main result of the present experiment was that the stimulating effect of chewing on performance, previously described in subjects chewing sequentially on both sides (Tramonti Fantozzi et al., 2017), could be observed mainly (Figures 3A,B) when this activity was performed on the hypotonic side, regardless of the clenching dominance of the subjects. This finding indicates that the positive reinforcement of sensorimotor trigeminal activity on cognitive efficiency is biased by the sign of the asymmetry observed in EMG activity during clenching. These observations suggest that a basal asymmetry in trigeminal input changes the excitability of ARAS’s structures, making the hypertonic side less responsive to the afferent input elicited by chewing and leading to less cortical arousal. Unfortunately, in the present experiment, the task-related mydriasis (an indicator of cortical arousal) associated to the matrices test was not evaluated and the mydriasis relative to the haptic task was not modified by 1 min of unilateral chewing. However, pupil size changes evaluated at rest after the end of chewing activity could represent a residual effects of ARAS activation during chewing. Values at T1 and at T2 showed significant differences with respect to T0, suggesting a persistent arousing effect of chewing (Figures 3C,D). In effects the changes in pupil size were larger when chewing occurred on the hypotonic side, at least when the pupil ipsilateral to the masticatory activity was considered. Apparently, this observation could be consistent with the hypothesis that chewing on the hypotonic side is more effective in activating ARAS structures. The lower sensitivity of the ARAS on the hypertonic side to the chewing-associated trigeminal stimulation could be attributed either to a ceiling effect, or to a loss of response to the incoming signals.

However, another hypothesis must be considered. A side-dependent effect of chewing was observed not only for performance parameters, but also for anisocoria, which indicates the existence of an imbalance in ARAS structures controlling arousal and pupil size. Anisocoria was reduced when chewing occurred on the hypotonic side, while increased following chewing on the hypertonic side (Figure 3E). These results were obtained since chewing on the hypotonic side elicited an increase in pupil size that was larger on the hypotonic (Figure 3D, continuous line) than on the hypertonic side (Figure 3C, continuous line), while chewing on the hypertonic side induced the opposite effect (compare dotted lines of Figures 3C,D). It has been previously shown, in subjects bearing an EMG asymmetry, that changes in mandible position and occlusal condition modified both anisocoria and cognitive performance, which were negatively correlated to each other (Tramonti Fantozzi et al., 2021b,c). Therefore, the higher efficacy of chewing on the hypotonic side in boosting cognitive performance, could be attributed to its higher effectiveness in reducing the imbalance in ARAS structures controlling arousal and pupil size. This reduction would lead to a more symmetrical cortical excitability and, as consequence, to an improved performance. There is indeed evidence that unilateral lesions in temporoparietal areas induced dramatic cognitive deficits that recover following a second, symmetric lesion (Lomber and Payne, 1996).

### Relations Between Changes in Performance, Pupil Size, and Anisocoria Following Chewing on the Hypertonic and Hypotonic Side

The results of correlative analysis were apparently consistent with both the hypotheses reported above. In fact, changes in both PI and SR were negatively correlated with changes in anisocoria, during chewing on the hypotonic (Figures 7A,B) but not on the hypertonic side (Figures 7C,D). However, during hypotonic side chewing positive correlations were observed between performance and pupil size changes, whatever pupil (at hypertonic or hypotonic side) was considered (Figures 5A,B, 6A,B). Yet, no correlation could be observed when chewing occurred on the hypertonic side (Figures 5C,D, 6C,D).

Overall, the effects of chewing on hypotonic side could be secondary to a rebalancing of ARAS structures, leading to anisocoria reduction, or else to an increased arousal level, expressed by a persistent pupil dilatation following chewing. Whatever the reason could be, in subjects with EMG asymmetry during clenching, an ARAS imbalance biases the stimulating effects of trigeminal sensorimotor activity. We may put forward the hypothesis that, following chewing on the hypotonic side, the arousing effects of trigeminal stimulation are enhanced by a rebalancing of ARAS structures, thus leading to a large increase in cognitive performance. At variance, following chewing on the hypertonic side, the arousing effect of trigeminal stimulation is partially or completely prevented by the simultaneous increase in ARAS imbalance.

It can be argued that the correlations between anisocoria, pupil size and performance changes observed in the present and in previous studies (De Cicco et al., 2014; Tramonti Fantozzi et al., 2021a,b,c) could be related to the constraining conditions characterizing pupil size recording, which may influence the subjective level of stress. However, the evaluation of cognitive performance was carried out in normal vision and without head restraining. It is therefore unlikely that these correlations arise from the limitations imposed by pupil size recording. Rather, changes in unrestrained measurements of performance can be predicted by changes in anisocoria measurements obtained in constrained conditions. Finally, despite the similarity of the pupil size recording procedures, the different studies addressing the correlation between performance and anisocoria changes have exploited different mandible positions and occlusal conditions. In the present report, the arches were always slight apart, but this was not the case in previous investigations, where they could be brought into contact either directly or through the interposition of a bite (De Cicco et al., 2016; Tramonti Fantozzi et al., 2021a,b, c).

Of course, the existence of a correlation between variables does not prove a causal relation between them: it could simply arise by a higher common source of association between the variables. It must be pointed out, however, that an occlusal correction able to reduce the EMG asymmetry during clenching also reduces anisocoria and improves performance. Therefore, the changes in EMG asymmetry are triggering both anisocoria and performance modifications. Although there is no definitive evidence about the direct effect that neural activities related to anisocoria exert on performance, it is likely that both of them are the expression of an imbalance in ARAS activity which is detrimental for cognition (Lomber and Payne, 1996); moreover there are at least two structures, the cholinergic and the noradrenergic systems which belong to ARAS, receive trigeminal signals and control both arousal and pupil size (Reimer et al., 2016; De Cicco et al., 2018; Peinkhofer et al., 2019).

### Stimulating Effects of Chewing on Cognitive Performance: The Locus Coeruleus Hypothesis

An important ARAS structure involved in cognitive control is the LC complex (Aston-Jones and Cohen, 2005; Grueschow et al., 2020). Many studies have underlined the strong covariation between its activity and pupil size, both in humans (Murphy et al., 2014) and animals (Rajkowski et al., 1994; Joshi et al., 2016). This link is attributable to the inhibition exerted by the LC on the Edinger-Westphal parasympathetic nucleus, which controls the iris constrictor and whose activity must be strongly reduced in order to allow pupil size increasing following activation of the weaker iris dilatator (Szabadi and Bradshaw, 1996; Wilhelm et al., 2001; Samuels and Szabadi, 2008). For these reasons, the pupil size is at present considered a proxy of LC activity (Mathôt, 2018). Within this structure the hyperactivity may trigger a cascade of molecular processes leading to neurodegenerative changes that spread from LC neurons to the rest of the brain (Weinshenker, 2018). It may well be that, as a preliminary step, an abnormally high activity in the LC leads to pathological alterations of neuronal responsiveness to the afferent input, which constrains the stimulating effects of trigeminal afferents. In this respect, the result of the present experiments indicates that the hyperactive side with its refractoriness to the stimulating effect of chewing is a dysfunctional element in the path linking the orofacial system to cognitive improvement. This finding is consistent with the observation that, in experiments where mandible position and occlusal condition were modified, changes in the hypertonic pupil size were the best predictor of performance changes (Tramonti Fantozzi et al., 2021b,c).

### Differences Between Right and Left Dominant Subjects

The results of the present experiments enlighten some differences in pupil size and performance-related parameters between right and left dominant subjects. In particular, the ER was larger in right dominant subjects. Since noradrenergic ascending projections are mainly ipsilateral (Berridge and Waterhouse, 2003; Wagner-Altendorf et al., 2019), this finding could be related to a larger noradrenergic tone impinging on the right hemisphere (Kruglikov et al., 1992), dominant for those spatial skills (Joseph, 1988) required in the matrices test. An excessive level of basal noradrenergic activity is in fact detrimental for performance, according to the “adaptive gain theory” proposed by Aston-Jones and Cohen (2005). Another between group difference was that the right and left dominant subjects differed in the left pupil size, while average right pupil size values of the two groups matched. Given the proposed relation between LC activity and pupil size (Rajkowski et al., 1994; Murphy et al., 2014; Joshi et al., 2016; Mathôt, 2018; Joshi and Gold, 2020), this puzzling observation suggests that the level of central noradrenergic activity could be kept constant on the right side, the one showing the larger noradrenergic tone (Kruglikov et al., 1992). In this perspective, asymmetries would develop following changes on left side, where LC activity may either increase or decrease. A larger sample of subjects, however, has to be investigated in order to confirm this hypothesis.

Right and left dominant subjects showed also some differences in the effects of unilateral chewing on these parameters. Among right, but not left dominant subjects, anisocoria (Figure 4E) was enhanced by hypertonic side chewing, due to a larger increase in pupil on the hypertonic side (Figure 4C). This larger increase in size of the hypertonic side pupil indicated that among right dominant subjects the ARAS structures on the hypertonic (right) side were more responsive to unilateral chewing with respect to those (left located) of left dominant subjects. Yet, hypertonic side chewing increased PI among left, but not right dominant subjects (Figure 4A). These data suggest that changes in performance following unilateral chewing are biased by the corresponding changes in anisocoria, rather than by refractoriness of the hypertonic side to chewing-related sensorimotor activity.

The larger increase in hypertonic side pupil observed after unilateral chewing among right dominant subjects could be related to a higher excitability of the right LC complex. In this respect, the right LC is also the main contributor to the higher noradrenaline content of the right hemisphere (Kruglikov et al., 1992). Moreover, it must be considered that left and right hemispheres differ in the modulation of sympathovagal autonomic balance. In particular, the right and left insular cortices appear to play a predominant role in establishing the sympathetic and the parasympathetic tone (Nagai et al., 2010, 2017), respectively. Moreover, the pupil diameter is correlated with the BOLD signal observed within the right insular cortex (DiNuzzo et al., 2019), a structure which receives noradrenergic afferents from the LC (Jones and Yang, 1985). Although the cortical and subcortical networks controlling pupil size are complex, it is likely that the differences between left and right dominant subjects reported above are related to the asymmetry (a) in norepinephrine content of hemispheres and (b) in the hemispheric control of the sympathovagal autonomic balance.

## Conclusion

In conclusion, the results of the present experiments highlight only minor differences between right and left dominant subjects, while indicate that the presence of an asymmetry in EMG activity during clenching constrains the stimulating effects of chewing on performance, which occur mainly when mastication is performed on the hypotonic side. These results could be attributed to a refractoriness of ARAS structures on the hypertonic side to sensorimotor trigeminal activation, or else to a change in the ARAS imbalance which is detrimental for cognitive performance and is reduced following chewing on the hypotonic side. It is possible that, chewing on the hypotonic side and hypertonic side, the arousing effects of trigeminal stimulation are enhanced and depressed by balancing and unbalancing ARAS structures, respectively. This would result in a larger increase in cognitive performance when chewing occurs on the hypotonic side. Although further investigations are necessary in order to definitively assess the precise ARAS structures involved in these phenomena, both animal and human studies point to the LC as a crucial hub in the path linking trigeminal afferents to cognitive control.

## Data Availability Statement

The raw data supporting the conclusions of this article will be made available by the authors, without undue reservation.

## Ethics Statement

The studies involving human participants were reviewed and approved by the University of Pisa Bioethical Committee. The patients/participants provided their written informed consent to participate in this study.

## Author Contributions

VDC, EC, and UF contributed to the conception and design of the study. MPTF, DDC, and DM organized the database. MPTF and DM performed the statistical analysis and wrote the first draft of the manuscript. DDC, Pd’A, LB, and UF wrote the sections of the manuscript. All authors contributed to manuscript revision, read, and approved the submitted version.

## Conflict of Interest

The authors declare that the research was conducted in the absence of any commercial or financial relationships that could be construed as a potential conflict of interest.

## Publisher’s Note

All claims expressed in this article are solely those of the authors and do not necessarily represent those of their affiliated organizations, or those of the publisher, the editors and the reviewers. Any product that may be evaluated in this article, or claim that may be made by its manufacturer, is not guaranteed or endorsed by the publisher.

## Footnotes

1
https://www.ibm.com/support/pages/downloading-ibm-spss-statistics-20
